# Green Extraction of Bioactive Compounds from Apple Pomace from the Cider Industry

**DOI:** 10.3390/antiox13101230

**Published:** 2024-10-14

**Authors:** Rosa Pando Bedriñana, Roberto Rodríguez Madrera, María Dolores Loureiro Rodríguez, Karelmar López-Benítez, Anna Picinelli Lobo

**Affiliations:** 1 Area of Food Technology, Regional Agrifood Research and Development Center (SERIDA), Carretera AS267, PK19, Villaviciosa, 33300 Asturias, Spain; rpando@serida.org (R.P.B.); rrodriguez@serida.org (R.R.M.); karelmar@serida.org (K.L.-B.); 2 Estación de Viticultura e Enoloxía de Galicia, Ponte San Clodio s/n, Leiro, 32428 Orense, Spain; maria.dolores.loureiro.rodriguez@xunta.gal

**Keywords:** polyphenols, triterpenic acids, ultrasound-assisted extraction, apple by-products

## Abstract

The cider-making industry in Asturias generates between 9000 and 12,000 tons of apple pomace per year. This by-product, the remains of the apple pressing, and made up of peel, flesh, seeds and stems, is a valuable material, containing substantial amounts of antioxidant compounds associated with healthy properties. Polyphenols such as dihydrochalcones and quercetin glycosides, and triterpenic acids, among which ursolic acid is a major compound, are the main antioxidant families described in apple pomace. The simultaneous recovery of those families has been accomplished by low frequency ultrasound-assisted extraction. Working extraction conditions were optimised by response surface methodology (RSM): time, 5.1 min; extractant composition, 68% ethanol in water; solid/liquid ratio, 1/75 and ultrasonic wave amplitude, 90%. This procedure was further applied to analyse those components in the whole apple pomace (WAP), apple peel (AP) and apple flesh (AF). On average, dry WAP contained almost 1300 µg/g of flavonols, 1200 µg/g of dihydrochalcones and 4200 µg/g of ursolic acid. These figures increased in the apple peel to, respectively 2500, 1400 and 8500 µg/g dry matter. Two linear multivariate regression models allowed the antioxidant activity of apple by-products to be predicted on the basis of their bioactive composition. The results derived from this study confirm the potential of industrial cider apple pomace as a source of high-value bioactive compounds, and the feasibility of the ultrasound-assisted extraction technique to recover those components in a simple and efficient way.

## 1. Introduction

Apples are one of the most highly consumed fruits worldwide. According to the FAO statistics, the global production of apples reached 95 M tons in 2022, of which 25–30% were used to make different apple derivatives, with apple juice concentrate as the main product [1,2]. In the European Union, where almost 60% of the world’s cider production and consumption occur, more than 1 M tons of apples were processed for the cider industry in 2018 [3]. The cider-making industry generates apple pomace as its main by-product, and this represents 25–30% of the initial weight of processed apples. This material, consisting of remains of peel, flesh, seeds and stems, contains substantial amounts of valuable compounds [4,5,6]. The phenolic profile of apple consists of flavanols (epicatechin and procyanidin oligomers), hydroxycinnamic acids (chlorogenic acid being the major one), flavonols (quercetin glycosides) and dihydrochalcones (phloridzin and phloretin 2′-xyloglucoside), whose relative concentrations depend on the apple variety and the stage of maturity [7,8,9]. Among the triterpenic acids, three major compounds are commonly identified in apples: ursolic, oleanolic and betulinic acids [10]. Most compounds present in the apple fruit remain in the apple pomace, so this material is a good source of phenolics and triterpenic acids, having a recognised bioactive potential [5].

Currently, apple pomace is used as a supplement in livestock feed, as a raw material to obtain pectin, or as biomass for energy production [5].

The use of apple pomace as a fortifying ingredient to increase the nutritional value of different types of foods has been proposed. Apple pomace, either directly or after minimal processing, has been successfully employed as an ingredient in the making of bakery, meat, confectionery and dairy products [11,12]. The research on the potential health risks associated with the ingestion of apple pomace is still limited and this requires more study. The presence of natural toxins, such as amygdalin, and phytosanitary products has been identified as a possible risk [13]. Regarding the content of cyanogenic compounds, amygdalin is located in the fruit seeds, with a concentration range of between 13.4 and 18.6 mg/g dry weight in cider apple seeds. These contents are reduced by two orders of magnitude in apple pomace [14]. Regarding the presence of pesticides and fungicides, current studies indicate that their levels in apples, and therefore in apple pomace, are below the tolerable limits established by law. Therefore, apple pomace as a food ingredient can be considered safe for human intake [15].

The extraction of bioactive components from apple pomace to be used in the food and pharmaceutical industries is another strategy that would add value to the cider industry while contributing to the reduction of waste [16].

The first challenge in recovering valuable molecules from complex materials is to free them from the matrix in which they are embedded and, secondly, to extract these molecules in an efficient and sustainable manner. This requires the application of techniques ensuring the highest extraction yield with a minimum impact on the quality of the final product. A number of novel techniques, which are alternatives to the conventional maceration or Soxhlet methods, have been proposed and critically compared [16,17]. Amongst these new techniques, ultrasound-assisted extraction can optimise the process parameters, thus reducing or eliminating the use of toxic organic solvents, while improving the extraction efficiency. Moreover, UAE offers clear advantages over other techniques, such as simplicity of use, versatility, flexibility, and low investment and energy consumption [18]. Currently, extraction assisted by ultrasound energy is being successfully applied for the recovery of bioactive components from different matrices, both at laboratory and industrial scales. The range of ultrasound frequency between 20 and 100 kHz—high power ultrasound—gives rise to cell wall disruption and enhanced mass transfer, thus allowing the extraction process to be achieved within minutes [19].

Different ultrasonic systems have been applied for the extraction of phenolic compounds from apple pomace. It is worth highlighting the studies carried out by Virot et al. [20] and Pingret et al. [21] using ultrasonic baths. The optimised factors were the ultrasonic energy, temperature (up to 40 °C) and sonication time (up to 45–55 min), further assayed and confirmed at pilot-scale. Both proposals reported that extraction assisted by ultrasound energy increased the recovery yield of total phenolic content by 20–30% compared to the conventional maceration method [20,21].

More recently, systems based on the use of ultrasonic probes have been evaluated for the extraction of polyphenols from apple pomace. The target variables for extraction optimization were TPC, antioxidant activity and dihydrochalcones [22,23]. The temperature (40/65/90 °C) affects the variables of interest, and the higher the temperature, the higher the TPC yield and the lower the antioxidant activity [22]. The ethanol:water ratio in the extractant mixture affects the recovery of dihydrochalcones, their content being the lowest when using 0% ethanol at the shortest time tested [23].

Triterpenic acids are promising secondary metabolites, having a wide variety of pharmacological properties including, among others, antimicrobial, antioxidant, anti-inflammatory and antimycotic, thus justifying the increasing attention they have been receiving in research and industry. Among them, ursolic acid is the most abundant triterpenoid in apples, followed by its isomeric compounds oleanolic and betulinic acids [6,10,24]. Because of their lipophilic nature, organic solvents like ethyl acetate, chloroform and ethanol are commonly used for extraction from different vegetal sources. Although classical methods such as maceration and Soxhlet and heat reflux extraction are usually reported for the extraction of triterpenic acids, other non-conventional techniques can be applied with good yield and recovery. For instance, UAE has been successfully applied for obtaining oleanolic and ursolic acids from medicinal herbs, leaves and pomegranate flowers and apple pomace [25,26]. Other strategies such as supercritical CO_2_ extraction have also been evaluated [27].

The aim of this study is to optimise an ultrasound-assisted method using a central composite design to simultaneously obtain total polyphenols and triterpenic acids from apple pomace. The method was then applied to analyse the profiles of polyphenols of low molecular mass and triterpenic acids in ten samples of apple by-products from the cider-making industry to evaluate their potential as a source of bioactive compounds. Furthermore, the corresponding fractions of apple peel and flesh were also analysed by HPLC-DAD.

## 2. Materials and Methods

### 2.1. Samples

Ten samples of apple pomace (40–50 kg each) were taken at different regional cider making establishments immediately after unloading the material from the presses. Each pomace was the result of a mixture of Asturian varieties included in the Asturian Cider Protected Designation of Origin of the 2021 harvest, after processing with pneumatic (8) or horizontal hydraulic (2) presses. The samples were transported to the SERIDA facilities and dried in a rotary oven at 60 °C for 48 h. Subsequently, a part of each apple pomace was divided into peel and flesh. Then, the different kinds of samples (whole apple pomace (WAP), apple peel (AP) and apple flesh (AF)) were milled and sieved (using a 1 mm sieve), stored under vacuum and protected from light until analysis.

### 2.2. Standards and Reagents

Polyphenol standards (chlorogenic acid, (-)-epicatechin, phloridzin, rutin, isoquercitrin, hyperin, and quercitrin) and triterpenic acids (uvaol, corosolic, betulinic, oleanolic, ursolic, and maslinic) were provided by Merck Life Sciences (Madrid, Spain). Avicularin and reynoutrin were provided by Cymit Quimica (Barcelona, Spain). Acetic acid was supplied by Panreac (Barcelona, Spain). The rest of the solvents and reagents (absolute ethanol, methanol, Folin–Ciocalteu reagent, ascorbic acid, 2,4,6-tris-(2-piridyl)-s-triazine (TPTZ), 2,2′-diphenyl-β-picrylhydrazyl (DPPH), ferric chloride and sodium acetate) were obtained from Merck Life Sciences (Darmstadt, Germany).

### 2.3. Optimization of the Extraction Method

A central composite design combined with response surface methodology was applied to optimise the extraction of total phenols and ursolic acid from apple pomace. The modelling consisted of 30 experiments including 6 central points, with 5 levels (−α, −1, 0, +1, +α, with α = 2). Four factors were studied: time (minutes), sample mass (g), ethanol:water proportion (%), and amplitude (%). The extractions were conducted in centrifuge tubes, with a fixed extractant volume of 30 mL, thermostated at 25 °C in a cool water bath, using a 7-mm titanium sonotrode. Each variable response was computed by means of a second-order polynomial model (Equation (1)), where Y represents the variable response, X_1–4_ are the independent factors under study, β_0_ is the offset term, β_i_, β_ii_, and β_ij_ are, respectively the first, second and interaction coefficients determined from the experimental results, and ε is the error term.


(1) $$Y\;=\;\beta_{0}+\sum_{i=1}^{4}\beta_{i}X_{i}\;+\sum_{i=1}^{4}\beta_{ii}X_{i}^{2}+\sum \sum_{i<j=1}^{4}\beta_{ij}X_{i}X_{j}+\varepsilon$$

Design Expert 7.0.0. (Stat-Ease, Inc., Minneapolis, MN, USA) was used to analyse the results of the optimization of the extraction method.

### 2.4. Extraction Method

An ultrasonic device (Hielscher UP200Ht, Hielscher Ultrasonics, Teltow, Germany) equipped with a 7-mm sonotrode was used. Around 0.4 g of sample (WAP, AP, AF) was extracted with 30 mL of ethanol:water (68:32) for 5.1 min, applying an ultrasonic wave amplitude of 90%. After that, the extract was centrifuged (20,000× *g* rpm, 5 min), filtered through a 0.45 µm-PVDF filter and analysed.

### 2.5. Total Phenols and Total Flavonoid Analyses

Total phenols content (TPC) was determined by the Folin–Ciocalteu method, according to the procedure described elsewhere [28]. The reaction was conducted in 10-mL volumetric flasks to which the different reactants were added in this order: 200 µL of sample, 5 mL of water, 250 µL of Folin–Ciocalteu reagent, 750 µL of 20% sodium carbonate, and water to reach the final volume. The absorbance was measured at 750 nm after 30 min at room temperature. The results are expressed as mg gallic acid/g dry weight.

Total flavonoids content (TFC) was determined by the aluminium chloride method, as described by Kim et al. [29], with slight modifications. A 500 µL aliquot of sample extract was added to a 5-mL volumetric flask containing 2 mL of distilled water and 150 µL of 5% NaNO_2_. After 5 min, 150 µL of 10% hexahydrated AlCl_3_ was added and mixed. Six minutes later, 1 mL of 1 M NaOH and 1.2 mL of water were added and thoroughly mixed. The absorbance was read at 510 nm. Results were expressed as mg rutin/g dry weight.

Both parameters were measured by using an Agilent Cary 60 UV-VIS spectrophotometer (Agilent, Santa Clara, CA, USA) equipped with an autosampler, in 1 cm disposable cuvettes.

### 2.6. Chromatographic Analyses

HPLC analyses of both polyphenolic compounds and triterpenic acids were performed by means of a Waters system equipped with a 717 plus automatic injector, a 600 pump, a 2998 diode array detector, a column oven (30 °C), and Empower 3 software. A Nucleosil 120 C_18_ (250 × 4.6 mm I.D., 3 µm) column from Macherey-Nagel (Teknokroma, Barcelona, Spain) was used. The elution solvents were aqueous 2% acetic acid (solvent A) and methanol (solvent B). Polyphenols were eluted at 0.80 mL/min according to the gradient previously reported and validated [7,30]. Triterpenic acids were isocratically eluted at 0.80 mL/min with 90% of solvent B. The injection volume was 50 µL. Quantification was performed by the external standard method at 210 nm (triterpenic acids), 280 nm (epicatechin and dihydrochalcones), 320 nm (chlorogenic acid) and 350 nm (flavonols). For those analytes where no standard was available, standards of the same family were used. Thus, dihydrochalcones were quantitated as phloridzin. The extracts were injected in duplicate, with the repeatability (RSD) always being less than 4%.

### 2.7. Antioxidant Activity Measurements

#### 2.7.1. DPPH Assay

The antiradical activity was determined by spectrophotometry in 1 cm disposable plastic cells, according to the method described elsewhere [7]. In brief, 40 µL of sample extracts or standards (ascorbic acid, in the range between 10–300 mg/L) were added to 1.460 mL of a DPPH solution (40 mg/L in methanol) and left to stand in the dark for 240 min. The absorbance at 515 nm was measured. The antioxidant activity was referred to as ascorbic acid (AA) equivalents [7].

#### 2.7.2. FRAP Assay

The working FRAP reagent was prepared freshly every day by mixing 2.5 mL of TPTZ (10 mM in 40 mM hydrochloric acid), 2.5 mL of ferric chloride (20 mM) and 25 mL of sodium acetate buffer (300 mM, pH 3.6). The FRAP assay was carried out at 37 °C, in 1 cm disposable plastic cells. FRAP reagent (900 μL) was mixed with 90 μL of water and 30 μL of apple pomace extract (diluted 1:5 with ethanol). After 120 min, the absorbance at 595 nm was measured. The antioxidant capacity of the apple pomace was expressed as AA equivalents [7].

Both assays were performed with the aforementioned Agilent Cary 60 equipment (Agilent, Santa Clara, CA, USA).

### 2.8. Statistical Analyses

ANOVA and Tukey’s test using a 95% confidence interval were applied to evaluate the effect of the type of sample on the contents of the components analysed. Linear multivariate regression analyses were performed to estimate the influence of the apple by-products’ composition on the antioxidant activities as measured by the DPPH and the FRAP in vitro methods. A free version of IBM SPSS Statistics v.29.02.0 was used. A forward selection method was applied to determine the independent variables that best predicted antioxidant activity.

For each model, its multivariate regression coefficient and the standard error of estimation were calculated. The percentage of antioxidant activity (AA) estimation was obtained for each sample according to Equation (2):

Estimation Percentage (%) = (AA_estimated_/AA_measured_) × 100 (2)

## 3. Results

### 3.1. Model Fitting

A central composite design was applied to optimise the extraction conditions to simultaneously obtain ursolic acid, the major triterpenic acid in apple, and total phenolic compounds. In all the experiments, a fixed extractant volume of 30 mL was used. Four independent factors at five levels were studied: extraction time (1, 2.5, 4, 5.5, and 7 min), sample mass (0.2, 0.4, 0.6, 0.8, and 1.0 g), ethanol:water proportion (40, 55, 70, 85, and 100%), and amplitude (60, 70, 80, 90, and 100%). The results are summarised in Table 1.

Under the experimental conditions of the proposed CCD, the contents of total phenolic compounds ranged between 3.07 and 9.42 mg gallic acid/g dry weight, obtained, respectively, with extractant mixtures containing 100% and 55% ethanol. Meanwhile, the area/mg ratios of ursolic acid, ranging between 5.02 and 1246.16, increased with increasing percentage of ethanol in the extractant mixture (Table 1). Mean values for total phenol content and ursolic acid area/mg ratio were 8.55 mg gallic acid/g and 1173.19 area/mg at the central point (coded variables 0). The axial points assayed for sample mass, ultrasonic amplitude and extraction time gave different results. On the one hand, the increase in sample mass produces a decrease in both dependent variables; on the other hand, an increase in the amplitude and extraction time results in higher values of both dependent variables (Table 1).

Table 2 shows the results of fitting the second-order polynomial models for the dependent variables. The ANOVA results indicated that both regression models were significant. Satisfactory determination coefficients were obtained for both variables. Adjusted R^2^ values were 0.915 (TPC) and 0.941 (ursolic acid), implying that the greatest percentage of the variance was explained by the respective models. Moreover, the difference between these and the predicted R^2^ values showed that the mathematical models were not overfitted. The resulting coefficients of variation (6.4% for TPC and 10.5% for ursolic acid) indicated the degree of precision with which the treatments are compared.

**Figure 1:**
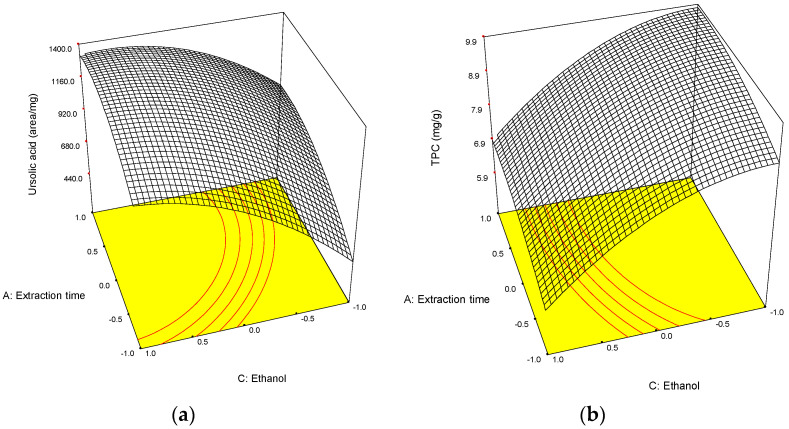
Response surfaces calculated from experimental data: (**a**) Ursolic acid content (area/mg ratio); (**b**) Total phenolic content (TPC, mg gallic acid/g dry weight). Mass sample: 0.4 g and amplitude of sonication: 90%.

As shown in Table 2, all of the factors analysed had a significant effect on the extraction of total polyphenols and ursolic acid, the ethanol content in the extractant mixture being the most influential, although with different effects. On the one hand, the extraction of ursolic acid was positively (β_3_ > 0) influenced by the ethanol content, as triterpenic acids are not soluble in water. In fact, its maximum extraction was obtained with an 85% ethanol (coded value: +1 on Table 1 and Figure 1a). On the other hand, the TPC, which includes families such as procyanidins, phenolic acids, flavonols and dihydrochalcones, having different solubilities in ethanol:water mixtures, was negatively affected by the ethanol content (β_3_ < 0, Table 2), reaching its highest value at 55% ethanol:water (coded value = −1, Figure 1b), as mentioned before. This result has already been observed for the extraction of phenolic compounds in apple pomace [20,23] and other matrices [31,32,33,34].

It is also worth highlighting the influence of the extraction time, showing a maximum between 4.0 and 5.5 min for both models (coded values 0, +1, Table 1), which reinforces the efficiency of the ultrasound extraction to recover components from different matrices. Previous reports on the use of ultrasonic probes to extract total phenols from apple pomace suggested longer extraction times than those tested in the present study [22,23].

Applying ultrasonic energy directly to the sample through high-power probes can enhance the yields for short extraction times [18]. Thus, based on previous experience in ultrasound-assisted extraction of polyphenols in dry beans of *Phaseolus vulgaris*, very short ranges of sonication times were evaluated, with satisfactory results [31]. The amplitude and sample mass were the factors that exerted least influence (Table 2).

Taking into account the mathematical models detailed in Table 2, the conditions that would maximize the concentrations of TPC and ursolic acid in the extract within the prediction interval (−1, +1) were optimised. Seven points, with a combined desirability of 0.984, which is considered optimal, were obtained. Among these points, all with minimal differences for each factor (<2%), the following conditions were selected: sample mass = 0.4 g, amplitude of sonication = 90%, extraction time = 5.1 min and ethanol concentration = 68%. For these conditions, the predicted values (Table 3) were higher than 98% of the maximum observed for each of them during the optimization process (Table 1), which can be considered as very satisfactory, taking into account that ethanol concentration showed opposing trends between the variables.

The optimised conditions were experimentally replicated in triplicate to validate the model. The values for TPC and ursolic acid are shown in Table 3. The results obtained were included within the predicted intervals for both variables, and the average accuracy of the values obtained against those predicted was 91% (TPC) and 98% (ursolic acid), so that the selected experimental conditions can be considered optimal to simultaneously extract phenolic compounds and triterpenic acids from apple pomace.

An exhaustive extraction of apple pomace was carried out to establish the method accuracy [35]. For this purpose, three samples of apple pomace were subjected to three consecutive extractions following the optimised protocol. The accuracy was estimated as % obtained in the first extraction. The values obtained showed an overall precision ranging from 90.3 to 93.0% for TPC and from 95.6 to 96.1% for ursolic acid, which shows the good accuracy and precision of the extraction method.

### 3.2. Antioxidant Composition of Apple By-Products

Table 4 summarises the contents of polyphenols and triterpenic acids found in apple pomace, peel and flesh, together with the antioxidant activity (DPPH and FRAP). The values for total polyphenols ranged in whole apple pomace (WAP) between 7.4 and 12.3 mg gallic acid/g dry weight, while those for total flavonoids varied between 14.3 and 25.0 mg rutin/g dry weight. These figures are nearly twice those observed in the apple flesh fraction (AF). The average values of those parameters in the apple peel (AP) fraction are almost double those observed in the WAP.

Similar trends can be observed for antioxidant activities. According to the DPPH assay, the antioxidant activity varied in the order AP > WAP > AF, whereas from the results of the FRAP assay there were no significant differences between the antioxidant activities of WAP and AP.

#### 3.2.1. Polyphenolic Compound Profile

The general profiles of apple by-products consisted of (-)-epicatechin, chlorogenic acid, dihydrochalcones and flavonols, in agreement with the results reported elsewhere for industrial apple pomace [7]. In Asturias, cider is made from a complex mixture of cider apple varieties in order to obtain a must with mild acidic sensory characteristics. The phenolic profile of typical Asturian cider apple varieties is made up of three families: firstly, chlorogenic acid and other hydroxycinnamic acids, which on average represent 55% of the phenolic composition of the must, followed by flavanols (epicatechin and procyanidin B2), representing up to 30%. Finally, dihydrochalcones (phlorizin and phloretin 2′-O-xyloglucoside), comprise the remaining 15% [36]. The general procedure of cider-making involves washing of apples, inspection to reject the rotten ones, milling and pressing. During the milling and pressing steps, apple pomace undergoes different degrees of oxidation, which may influence the phenolic composition of this by-product. Oxidation of apple decreases the concentration of polyphenols in the resulting juice, particularly that of flavan-3-ols. These compounds remain attached to the apple cell walls, linked to xyloglucans and pectins, and so are not easily extractable [37,38].

The AP fraction has the highest content of flavonols. They represent between 48.9 and 79.2% of the polyphenolic composition analysed by HPLC, followed by the group of dihydrochalcones (12.1–44.9%). Flavonols are located almost exclusively in the apple peel [8,39,40]; they slowly diffuse into the juice during pressing so that flavonols are found almost entirely in apple by-products [37]. Hyperin (quercetin-3-galactoside), ranging between 600 and 1032 µg/g is the major component in this family, followed by avicularin (quercetin-3-*O*-α-L-arabinofuranoside), in agreement with previous reports on apple and apple pomace composition [7,8].

As seen in Table 4, the AF presented significantly lower contents of all the compounds analysed, except for chlorogenic acid, whose concentrations were similarly distributed among the different apple tissues. In the AF fraction, dihydrochalcones are the predominant group of polyphenols analysed by HPLC (36.6–72.5%), followed by flavonols (8.8–55.4%), and chlorogenic acid (5.6–16.4%).

Flavonols and dihydrochalcones were the main phenolic families found in all the apple by-product samples (Table 4). This result is interesting since phloridzin and flavonols are very stable upon drying and long-term storage, making apple pomace a promising material for value-added purposes [41]. Moreover, these two phenolic families are also stable under gastrointestinal pH conditions, suggesting their potential bioaccessibility [42].

#### 3.2.2. Triterpenic Acid Profiles

Triterpenic acids are abundant in various plants and occur in the free acid form or as aglycones for triterpenoid saponins. These components are predominant in waxes from apple peel, where they protect the fruit against biotic and abiotic stress factors [24,43]. The profile of triterpenic acids varies between commercial and ancient apple varieties; for instance, the skin of old cultivars is characterised by the presence of maslinic, annurcoic and pomaceic acids, while commercial ones show higher contents of oleanolic and ursolic acids [44]. Due to their localisation in the apple peel, apple pomace is a valuable source of these compounds [24].

Three terpenic acids have been observed in cider industry apple pomace, these being ursolic oleanolic and corosolic acids. The AP fraction presents significantly higher contents than WAP and AF (Table 4). Ursolic acid is the major component, representing between 64 and 72% of the total triterpenic acids in the three cider apple by-products studied. Its isomer, oleanolic acid, is also present in all the matrices studied, constituting between 24 and 35% of this family of compounds. Finally, corosolic acid is a residual component, absent in 8 of the 10 samples of the AF fraction.

The concentration of these compounds depends on the apple variety, harvest maturity, and rootstocks [10,43,44,45]. In the WAP samples analysed, the sum of triterpenic acids varied between 4933 and 7011 µg/g dry weight (d.w.), a range that in the AP group rises to 10,094 and 13,673 µg/g d.w. These figures are similar to those observed by Woźniak et al. [27] in apple pomace from the juice industry, but higher than the contents described by Andre et al. [10]. In their study characterizing the triterpene profiles of 109 apple varieties, these authors reported concentrations in the skin ranging from 75 to 5987 µg/g d.w. for ursolic acid, and from 80 to 1425 µg/g d.w. for oleanolic acid.

### 3.3. Antioxidant Activity and Composition

The DPPH scavenging assay and the ferric reducing power assay (FRAP) have been widely used for measuring the antioxidant activity of foods, beverages and by-products from the agrifood industries. Many previous studies have reported the antioxidant activity of apples and apple pomace in association with its phenolic composition [4,7,24,39,45], but only a few analysed the antioxidant activity of triterpenic acids [4,24,46]. In the present study, the combined effect of phenolic compounds and triterpenic acids on the antiradical activity (DPPH method) and reducing power (FRAP method) is studied.

Two linear multivariate regression models have been obtained to evaluate the influence of the bioactive components on the antioxidant activity (either DPPH or FRAP measurements) of the apple by-products extracts. Table 5 summarises the results of the models.

The model predicting the antioxidant activity by DPPH testing includes three phenolic compounds: an unidentified dihydrochalcone, a flavonol (rutin + isoquercitrin), and chlorogenic acid. It presents a satisfactory multiple regression coefficient (R^2^_adjusted_: 0.955) and a standard error of estimation of 1.0577. The model allowed a good prediction of the antioxidant activity of all the samples, with a mean prediction percentage of 101.5%. Only two extracts, belonging to the AF fraction, showed discordant prediction values (126.2 and 68.3%).

Again, a dihydrochalcone and a flavonol are the independent variables selected for the construction of the prediction model of the antioxidant activity measured by the ferric reduction assay (FRAP). It is interesting that oleanolic acid also participates in this model (Table 5). The FRAP model also presents satisfactory multivariate correlation coefficients (R^2^_adjusted_: 0.968) and standard estimation error (1.199). Unlike what was observed in the case of measurements with the DPPH test, the prediction ability of the FRAP regression model fails with most samples of the AF fraction. This may be due to a limitation in the quantification capacity of the method itself, since the lowest values of antioxidant activity are observed in this set of samples (Table 4).

The selected phenolic predicting variables are usually associated with the antioxidant capacity of apple by-products [4,7]. Dihydrochalcones and flavonoids exhibit their antioxidant capacity due to their ability to interact with oxidative species transferring an electron or a hydrogen atom. The activity of these molecules is influenced by structural features such as the presence of hydroxyl groups, resonance delocalisation of the phenolic radical and steric hindrance derived from voluminous glycoside groups substituting hydrogen in the aromatic rings [47,48].

Oleanolic acid contributed to the antioxidant activity of apple extracts in a smaller proportion than the phenols involved in the mathematical model (Table 5). Triterpenic acids exhibit lower antiradical and reducing activities than polyphenols, according to the results obtained by Grigoras et al. [4]. Enriched fractions of apple pomace extracts in ethyl acetate—containing mainly flavonoids—showed increased antioxidant activities compared to the crude extracts, whereas the heptane fraction, consisting mainly of triterpenic acids, presented antioxidant activities slightly reinforced in comparison to the crude extracts. Similar findings were reported by Cefarelli et al. [46] by comparing the antiradical activities (DPPH test) of pure components isolated from Annurca apples. However, triterpenic acids show strong inhibiting power when assayed for NO-scavenging activity and determination of thiobarbituric acid reactive substances (TBARS).

## 4. Conclusions

The results obtained in the present study provide information relating to the potential use of apple pomace from the cider industry for the extraction of compounds of high biological value. Although the AP fraction is enriched in polyphenols and triterpenic acids compared to WAP, the latter still has a high potential for use due to its high content of bioactive compounds. The economical and sustainable valorisation of cider apple pomace requires the optimisation of an efficient extraction technique and the subsequent scaling up to the industrial dimension. In this regard, a method like the one proposed in this study, based on the use of ultrasound and ethanol/water as the extracting solvent, could be a good alternative for recovering these biomolecules. The next stage within a by-product valorisation strategy includes the development of extraction processes on a pilot scale.

## Figures and Tables

**Table 1:** Real and coded values for the factors in the central composite design and experimental results obtained for ursolic acid and total phenolic compounds (TPC).

	Independent Factors				Response Variables	
	X1 (Time, min)	X2 (Mass, g/30mL)	X3 (Solvent, % ethanol)	X4 (Amplitude, %)	Ursolic Acid (Area/mg)	TPC (mg Gallic Acid/g Dry Weight)
1	2.5 (−1)	0.4 (−1)	55 (−1)	70 (−1)	242.59	7.69
2	5.5 (+1)	0.4 (−1)	55 (−1)	70 (−1)	760.81	9.11
3	2.5 (−1)	0.8 (+1)	55 (−1)	70 (−1)	235.62	7.78
4	5.5 (+1)	0.8 (+1)	55 (−1)	70 (−1)	331.61	8.29
5	2.5 (−1)	0.4 (−1)	85 (+1)	70 (−1)	1206.62	5.77
6	5.5 (+1)	0.4 (−1)	85 (+1)	70 (−1)	1188.93	5.72
7	2.5 (−1)	0.8 (+1)	85 (+1)	70 (−1)	1159.67	4.90
8	5.5 (+1)	0.8 (+1)	85 (+1)	70 (−1)	1209.29	5.69
9	2.5 (−1)	0.4 (−1)	55 (−1)	90 (+1)	446.61	8.53
10	5.5 (+1)	0.4 (−1)	55 (−1)	90 (+1)	842.24	9.42
11	2.5 (−1)	0.8 (+1)	55 (−1)	90 (+1)	376.27	8.70
12	5.5 (+1)	0.8 (+1)	55 (−1)	90 (+1)	455.52	9.15
13	2.5 (−1)	0.4 (−1)	85 (+1)	90 (+1)	1231.74	5.68
14	5.5 (+1)	0.4 (−1)	85 (+1)	90 (+1)	1246.13	6.42
15	2.5 (−1)	0.8 (+1)	85 (+1)	90 (+1)	1246.16	5.53
16	5.5 (+1)	0.8 (+1)	85 (+1)	90 (+1)	1226.05	6.05
17	1.0 (−2)	0.6 (0)	70 (0)	80 (0)	349.25	6.17
18	7.0 (+2)	0.6 (0)	70 (0)	80 (0)	1180.45	9.38
19	4.0 (0)	0.2 (−2)	70 (0)	80 (0)	1235.57	9.41
20	4.0 (0)	1.0 +2)	70 (0)	80 (0)	1165.03	7.84
21	4.0 (0)	0.6 (0)	40 (−2)	80 (0)	5.02	8.96
22	4.0 (0)	0.6 (0)	100 (+2)	80 (0)	1173.05	3.07
23	4.0 (0)	0.6 (0)	70 (0)	60 (−2)	919.83	8.25
24	4.0 (0)	0.6 (0)	70 (0)	100 (+2)	1199.66	9.06
25	4.0 (0)	0.6 (0)	70 (0)	80 (0)	1093.69	8.34
26	4.0 (0)	0.6 (0)	70 (0)	80 (0)	1185.21	8.21
27	4.0 (0)	0.6 (0)	70 (0)	80 (0)	1221.85	8.37
28	4.0 (0)	0.6 (0)	70 (0)	80 (0)	1107.48	8.36
29	4.0 (0)	0.6 (0)	70 (0)	80 (0)	1238.02	8.98
30	4.0 (0)	0.6 (0)	70 (0)	80 (0)	1192.89	9.02

**Table 2:** Statistics for models constructed for target variables in terms of coded values.

	TPC (mg Gallic Acid/g Dry Weight)		Ursolic Acid (Area/mg)	
Model		***		***
Intercept	8.39		1172.48	
β_1_-Time	0.49	***	101.59	***
β_2_-Mass	0.22	**	−44.44	**
β_3_-Ethanol	−1.45	***	348.31	***
β_4_-Amplitude	0.26	**	53.97	**
β_12_	-	-	−44.11	*
β_13_	-	-	−66.43	**
β_23_	-	-	53.81	**
β_11_	−0.28	***	−130.62	***
β_33_	−0.72	***	−153.35	***
β_44_	-	-	−35.67	*
Lack of Fit		n.s.		n.s.
R^2^		0.933		0.961
Adj R^2^		0.915		0.941
Predicted R^2^		0.816		0.864
C.V. %		6.4		10.5

(***) *p* < 0.01; (**) *p* < 0.05; (*) *p* < 0.10; (n.s.) no significant.

**Table 3:** Validation of model (mass: 0.4 g, time: 5.1 min; amplitude of sonication: 90%; ethanol: 68%) under conditions of reproducibility.

	TPC (mg Gallic Acid/g Dry Weight)	Ursolic Acid (Area/mg)
Prediction	9.26	1237.00
95% CI low	8.85	1146.00
95% CI high	9.66	1327.44
95% PI low	8.18	1015.40
95% PI high	10.33	1457.73
Real samples (*n* = 3)	8.43	1214.20
Repeatability, r (RSD%)	2.33	2.70

TPC: Total phenolic contents. CI: Confidence interval. PI: Prediction interval; RSD: Relative standard deviation.

**Table 4:** Profiles of phenolic compounds, triterpenic acids, and antioxidant activity of whole apple pomace and fractions of apple peel and apple flesh (*n* = 10).

	Whole Apple Pomace				Apple Peel				Apple Flesh			
	Mean	SD	Max	Min	Mean	SD	Max	Min	Mean	SD	Max	Min
Total phenols (mg gallic acid/g dry weight)	9.9b	1.5	12.3	7.4	15.1c	2.3	17.5	11.0	4.8a	1.5	7.5	3.4
Total flavonoids (mg rutin/g dry weight)	18.2b	3.4	25.0	14.3	32.0c	4.6	37.7	22.2	7.2a	2.3	13.3	5.4
Polyphenols (µg/g dry weight)												
Sum of HPLC polyphenols	2733b	443	3459	2027	4161c	701	5175	2736	1175a	395	2035	739
(-)-Epicatechin	79b	31	120	31	87b	38	161	32	29a	10	50	19
Chlorogenic acid	162a	52	252	84	134a	45	201	62	132a	35	184	85
Dihydrochalcone-1	79b	25	110	27	119c	43	181	27	44a	12	64	29
Phloretin 2′-Xyloglucoside	196b	79	321	50	255b	93	408	57	110a	47	188	51
Phloridzin	930b	237	1290	521	1064b	413	1652	248	529a	117	700	335
Hyperin	399b	72	518	279	803c	160	1032	600	90a	104	330	18
Rutin + Isoquercitrin	228b	40	305	184	449c	82	575	350	50a	58	189	12
Reynoutrin	118b	16	153	103	229c	37	290	185	32a	33	113	7
Avicularin	342b	48	435	275	648c	108	788	483	88a	90	309	26
Quercitrin	201b	28	250	147	371c	60	476	301	69a	52	186	17
Triterpenic acids (µg/g dry weight)												
Sum of triterpenic acids	5913b	749	7011	4933	11881c	1282	13673	10094	1576a	1351	4656	406
Corosolic acid	169b	41	232	98	323c	69	446	206	20a	43	102	nd
Oleanolic acid	1525b	216	1891	1238	3021c	395	3818	2540	453a	327	1184	146
Ursolic acid	4219b	546	5064	3529	8537c	921	9915	7294	1103a	986	3369	261
Antioxidant activity (mg Ascorbic acid/g dry weight)												
DPPH	9.7b	2.6	12.7	5.5	15.2c	1.9	17.2	10.9	4.2a	1.1	7.0	3.2
FRAP	10.7b	1.1	12.2	9.0	17.9b	2.1	20.1	13.6	2.0a	0.6	3.5	1.5

SD: Standard deviation; Max: Maximum; Min: Minimum. Different letters in the same row indicate significant differences at *p* < 0.05, nd, not detected.

**Table 5:** Parameters of linear multivariate regression models to determine the antioxidant activity of apple by-products (WAP, AP, AF) with respect to their phenolic and triterpenic acid contents.

Dependent Variables	Regression Model	Coefficients	Model Results
DPPH	β_0_	−0.398	R^2^: 0.959 R^2^_adjusted_: 0.955
	Dihydrochalcone-1	46.893	Standard estimation error: 1.0577
	Rutin + Isoquercitrin	18.131	Estimation (%)
	Chlorogenic acid	13.642	Mean: 101.5
			SD: 12.17
			Maximum: 126.2
			Minimum: 68.3
FRAP	β_0_	−2.053	R^2^: 0.972 R^2^_adjusted_: 0.968
	Rutin + Isoquercitrin	16.367	Standard estimation error: 1.199
	Phloridzin	5.153	Estimation (%):
	Oleanolic acid	2.372	Mean: 103.7
			SD: 31.06
			Maximum: 190.6
			Minimum: 37.7

## Data Availability

Data is contained within the article.
